# A Reference Hand-Book of the Medical Sciences

**Published:** 1886-06

**Authors:** 


**A Reference Hand-book of the Medical Sciences,** embracing the entire range of Scientific and Practical Medicine and Allied Science, by various writers. Illustrated by chromo-lithographs and wood engravings. Edited by ALBERT H. BUCK, M. D. Vol. II. New York: Wm. Wood & Co.

The second volume of this magnificent work more than sustains the high expectation with which it was looked forward to, and the announcement of its publishers that it is their aim to make it "the most practical and generally useful work to all classes of the profession" is being fairly fulfilled. The publishers deserve the hearty support of the profession. Those of our readers who have received the two volumes now issued are, we are sure, more than satisfied with them, and we would recommend those who have not yet subscribed to do so at once.

